# Delays in treatment initiation for patients with relapsing-remitting multiple sclerosis—A nationwide population-based study

**DOI:** 10.1177/20552173251360358

**Published:** 2025-08-04

**Authors:** Maiju Savolainen, Matias Viitala, Katariina Kuutti, Hanna Kuusisto, Ilkka Rauma, Mervi Ryytty, Johanna Krüger, Päivi Hartikainen, Marja Niiranen, Jukka Saarinen, Merja Soilu-Hänninen, Sini M Laakso

**Affiliations:** Department of Neurology, Brain center, Helsinki University Hospital and Clinical Neurosciences, University of Helsinki, Helsinki, Finland; StellarQ Ltd, Turku, Finland; Clinical Neurosciences, Neurocenter, University of Turku and Turku University Hospital, Turku, Finland; Department of Neurology, 60670 Tampere University Hospital, Tampere, Finland; Department of Health and Social Management, University of Eastern Finland, Kuopio, Finland; Neural Medicine Responsibility Sector, Department of Sensory, Neural, and Musculosceletal Medicine, Neurocenter Finland, Tampere Brain and Mind, Wellbeing Services County of Pirkanmaa, 60670 Tampere University Hospital, Tampere, Finland; Faculty of Medicine and Health Technology, Tampere University, Tampere, Finland; Research Unit of Clinical Medicine Neurology and Oulu University Hospital, Neurocenter, Neurology and MRC, 6370 University of Oulu, Oulu, Finland; Neuro Center, Neurology, 60650 Kuopio University Hospital, Kuopio, Finland; Neurology Departments, 86663 Wellbeing Services County of Ostrobothnia, Vaasa, Finland; Clinical Neurosciences, Neurocenter, University of Turku and Turku University Hospital, Turku, Finland; Department of Neurology, Brain center, Helsinki University Hospital and Clinical Neurosciences, University of Helsinki, Helsinki, Finland

**Keywords:** Multiple sclerosis, relapsing-remitting, disease-modifying therapies, treatment delay, patient refusal, registry-based study

## Abstract

**Background:**

Early disease-modifying therapy (DMT) improves outcomes in patients with relapsing-remitting multiple sclerosis (pwRRMS), but reasons for delayed or absent initiation are unclear.

**Objective:**

To investigate reasons and trends for delayed or absent DMT initiation among Finnish pwRRMS.

**Methods:**

A nationwide retrospective study using the Finnish MS Registry identified 2363 pwRRMS diagnosed between 2010 and 2019 in the participating centers. Patients never receiving DMT or starting >2 years post-diagnosis were compared to those initiating DMT within a year of diagnosis.

**Results:**

We identified 193 pwRRMS who never started DMT, 88 had delayed initiation over 2 years, and 1944 started within a year. The no/delayed DMT group was older at diagnosis (mean 38.7 vs 35.2 years, *p* < 0.001). Corticosteroid-treated relapses were more frequent among early initiators. Optic neuritis was more common in patients with delayed or no DMT. Treatment refusal was the primary reason for delayed/no DMT (35.6%), with 68% of refusers never starting. From 2010to 2019, delayed/no DMT initiation (*p* = 0.007) and treatment refusal (*p* = 0.004) decreased significantly.

**Conclusion:**

Delayed or absent DMT initiation is linked to older age, optic neuritis, disease inactivity, and treatment refusal, which declined over time, likely due to expanded DMT options.

## Introduction

Multiple sclerosis (MS) is the most common progressive neurological disease in young adults and the third leading cause of disability pensions among 16- to 44-year-olds in Finland.^1^ While MS prevalence has increased steadily over the past five decades, a notable rise in incidence occurred only during the 1990s.^2,3^ Currently, an average of 10 individuals per 100,000 are diagnosed with MS annually in Finland.^4^

Early initiation of treatment in patients with relapsing-remitting MS (pwRRMS) has been associated with a reduced risk of disability progression and lower likelihood of transition to secondary progressive MS (SPMS).^5^ For instance, initiating glatiramer acetate or interferon beta within 5 years of disease onset decreases the probability of converting to SPMS.^6^ Similarly, starting the first disease-modifying treatment (DMT) within 1 year of diagnosis significantly reduces the risk of functional decline compared to later initiation.^7^ Early initiation of high-efficacy DMTs within two years of onset has also been linked to better long-term outcomes.^8^

Despite these benefits, not all MS patients commence DMTs. In Finland, 34% of MS patients under specialized care were without a DMT in 2018.^9^ A multicenter French study suggested that less severe MRI findings and lower disability scores correlate with the absence of DMT initiation.^10^ The reasons for delayed or absent DMT initiation remain unclear but may include patient refusal, family planning, or perceived lack of disease activity. Understanding this patient population is crucial for improving MS care. This study aimed to investigate the frequency and reasons for delayed or absent DMT initiation in a nationwide cohort of pwRRMS in Finland.

## Methods

### Study setting

This is a retrospective observational study. The study population was derived from six Finnish wellbeing service counties with inclusion criteria of all clinically confirmed pwRRMS diagnosed between 2010 and 2019. RRMS is diagnosed and DMTs initiated in Finland solely in the public health care, thus rendering the study population-based for the participating regions. The included wellbeing service counties were Helsinki and Uusimaa, Southwest Finland, Northern Ostrobothnia, Vaasa, Northern Savonia, and Pirkanmaa.^9^

The patients with treatment delay of more than 2 years after diagnosis or no DMT initiation were compared to patients with treatment initiation within the first year after diagnosis. Registry data regarding the study cohort in the Finnish MS Registry was checked by chart review and corrected for missing data by expert MS neurologists from the central or university hospitals of the participating regions. Date of MS diagnosis was considered as index date for study patients. Partial dates were imputed to the midpoint of the respective month or year. Baseline Expanded Disability Status Score (EDSS) was defined as the recorded EDSS closest to the MS diagnosis date between 1 year prior and 1 month after the index date, however, EDSS values within a month from relapse were not included. Patients with a diagnosis of primary progressive MS or discrepancies in the timing of MS diagnosis were excluded from the study.

The Finnish MS Registry is a system developed jointly by the software company StellarQ and the expert group of the Finnish Neurological Association, and it is in use at all university hospitals and several central hospitals.^9^ The Finnish MS Registry was implemented in 2014. The registry includes structured data on key parameters for MS treatment: patient's age and sex, date of the diagnosis and its certainty level and subclass, medication history including start and end dates, possible reasons for medication absence, potential medication side effects, monitoring of the EDSS disability level development, relapses and the treatments given for them, as well as imaging results.

### Clinical parameters collected

Data on age at MS onset symptom, age at diagnosis, sex, EDSS points recorded at the time of diagnosis, annualized relapse rate (ARR) 1 year prior to MS diagnosis, time from diagnosis to first treatment initiation and reasons for delayed treatment or no treatment initiation were collected from the Finnish MS Registry.

### Ethical considerations

The study was approved by the Finnish Institute of Health and Welfare (THL/2058/14.02.00/2022). Data utilization was thus granted through Finnish secondary data use process. According to Finnish legislation, the approval of an ethical committee nor informed consent from patients was required as the study was a non-interventional registry study in which patients were not directly contacted or involved in data collection. The data was stored and analyzed in a pseudonymized format in the Academic data secure environment managed by Helsinki University Hospital.

### Statistical analysis

Descriptive statistics for demographics and clinical variables included means, standard deviations, medians, and quartiles for continuous variables, and counts and proportions based on non-missing data for categorical variables. In addition, the proportion of missing data was reported. Group comparisons for continuous variables were performed using the Student's *t*-test or Wilcoxon rank-sum test depending on the normality of data for compared groups. Fisher's exact test was used to compare categorical variables. *p*-Value adjustment was done using Benjamini & Hochberg method to control the False Discovery Rate. Trend analysis for reasons for no DMT initiation or delayed DMT initiation were done using Mann–Kendall trend test. In all analyses, two-tailed or corrected *p*-values under 0.05 were considered as significant. Data analysis and visualization were performed on pseudonymized data using RStudio (Version 2021.09.1 or newer).

## Results

### Demographic and clinical differences between early DMT initiators and delayed or no DMT use subgroups

**Figure 1.**
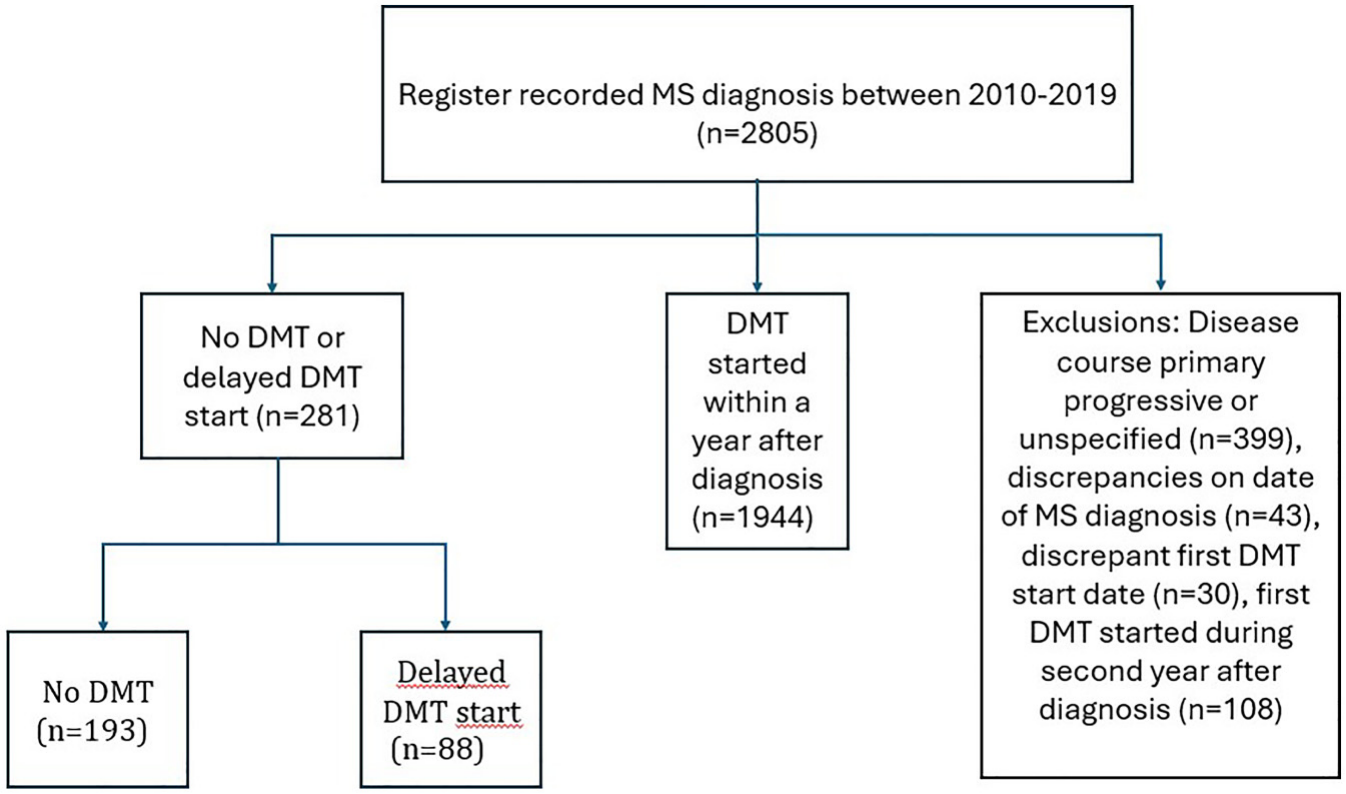
Flowchart of study population selection and categorization based on DMT usage. There were altogether 2805 patients with a confirmed MS diagnosis between 2010 and 2019 in the Finnish MS register. Of these, patients with no DMT use ever recorded (*n* = 193) or with a delayed initiation of more than 2 years from diagnosis (*n* = 88) were compared to patients who had initiated a DMT within 1 year after diagnosis (*n* = 1944). Patients were excluded based on disease course other than relapsing-remitting, discrepancies in data on date of diagnosis or DMT start and those that had started their first DMT during the second year after diagnosis (MS: multiple sclerosis; DMT: disease-modifying treatment).

Our registry data contained 281 pwRRMS who had never initiated a DMT (*n* = 193) or for whom the initiation was delayed by at least 2 years from diagnosis (*n* = 88). As the comparator group, there were altogether 1944 pwRRMS who had initiated a DMT within a year from diagnosis during the study period 2010–2019 in the participating centers (Figure 1). Patients with delayed or no DMT initiation were significantly older at diagnosis than those with an early DMT initiation (mean 38.7 vs. 35.2 years, respectively, *p* < 0.001) and had longer onset symptom-to-diagnosis times (median 4.4 vs. 1.0 years, *p* < 0.001; Table 1). The ARR was also lower in this group compared to early initiators (mean 0.7 vs. 1.1, respectively, *p* < 0.001), whereas onset symptoms presented at a similar age in both groups and EDSS scores did not differ significantly between groups. There was no statistically significant difference between men and women.

**Table 1.** Characteristics of patients with early versus delayed or no initiation of disease-modifying therapy for relapsing-remitting multiple sclerosis.

	DMT started within a year after MS diagnosis (*n* = 1944)	No DMT or delayed DMT start (*n* = 281)	*p*-Value
Sex (female); *n* (%)	1406 (72.3%)	207 (73.7%)	NS
Age variables; Mean (SD)			
Age at MS onset (years)	32.5 (9.66) [8%]	32.2 (10.34) [6%]	NS
Age at MS diagnosis (years)	35.2 (10.13) [0%]	38.7 (11.72) [0%]	<0.001***
Age at data cut-off (years)	42.7 (10.43) [0%]	47.2 (11.41) [0%]	<0.001***
Time since MS onset to MS diagnosis (years); Median (Q1–Q3)	1.0 (0.4–3.2) [8%]	4.4 (1.2–10.4) [6%]	<0.001***
ARR 1 year prior MS diagnosis; Mean (SD)	1.1 (0.73) [0%]	0.7 (0.62) [0%]	<0.001***
EDSS at MS diagnosis (0–10); Median (Q1–Q3)	1.5 (1.0–2.0) [64%]	1.0 (0.0–2.0) [33%]	NS
Cortisone-treated relapses one year prior to diagnosis (Yes); *n* (%)	665 (34.2%)	48 (17.1%)	<0.001***
Onset symptoms; *n* (%)			
Optic neuritis	417 (21.5%)	87 (31.0%)	0.005**
Brainstem syndrome	300 (15.4%)	42 (14.9%)	NS
Cerebellar syndrome	358 (18.4%)	42 (14.9%)	NS
Muscle weakness due to injury of the motor tract	296 (15.2%)	31 (11.0%)	NS
Sensory disturbance	844 (43.4%)	116 (41.3%)	NS
Other/unknown	293 (15.1%)	29 (10.3%)	NS
Multifocal symptoms; *n* (%)	301 (15.5%)	36 (12.8%)	NS

MS: multiple sclerosis; DMT: disease-modifying therapy; ARR: annualized relapse rate; EDSS: Expanded Disability Status Scale; NS: non-significant. Percentages are based on non-missing data. Patients could have had more than one onset symptom. Asterisks indicate statistical significance for comparison between early DMT and no/delayed DMT groups (*p < 0.05, **p < 0.01, ***p < 0.001).

### Clinical differences between no DMT initiation and delayed DMT initiation subgroups

We then compared pwRRMS who had never initiated a DMT to those for whom the initiation was delayed by at least 2 years from diagnosis. Lifetime lack of a DMT was associated with older age compared to those with delayed initiation at both disease onset (33.8 years vs 28.5 years, respectively; *p* < 0.001) and at diagnosis (41.2 years vs 33.4 years, respectively; *p* < 0.001; Table 2). For both groups, the majority had been diagnosed during 2010–2014 and a smaller proportion, approximately 30%, during 2015–2019. However, disease severity as recorded by ARR 1 year prior to MS diagnosis and EDSS at MS diagnosis were similar for both the patients with lifetime lack of DMT and those with delayed DMT initiation.

**Table 2.** Demographics of patients with no DMT initiation recorded compared to those with initiation after 2 years from diagnosis.

	No DMT (*n* = 193)	Delayed DMT (*n* = 88)	*p*-Value
Sex (female); *n* (%)	146 (75.6%)	61 (69.3%)	NS
Age variables; mean (SD)			
Age at MS onset (years)	33.8 (10.88) [4%]	28.5 (7.85) [9%]	0.001**
Age at MS diagnosis (years)	41.2 (11.83) [0%]	33.4 (9.52) [0%]	<0.001***
Age at data cut-off (years)	49.3 (11.76) [0%]	42.8 (9.21) [0%]	<0.001***
Time since MS onset to MS diagnosis (years); median (Q1–Q3)	5.3 (1.5–12.6) [4%]	3.0 (0.9–7.0) [9%]	0.010**
ARR 1 year prior MS diagnosis; mean (SD)	0.7 (0.60) [0%]	0.7 (0.67) [0%]	NS
EDSS at MS diagnosis (0–10); median (Q1–Q3)	1.0 (0.0–2.0) [30%]	1.8 (0.0–2.0) [41%]	NS
Diagnosis year interval; *n* (%)			NS
2010–2014	118 (61.1%)	65 (73.9%)	
2015–2019	75 (38.9%)	23 (26.1%)	
Time since diagnosis to first DMT; median (Q1–Q3)	NA (NA-NA) [100%]	4.8 (2.6–6.6) [0%]	
Cortisone-treated relapses 1 year prior to diagnosis (Yes); *n* (%)	36 (18.7%)	12 (13.6%)	NS
Onset symptoms; *n* (%)			
Optic neuritis	65 (33.7%)	22 (25.0%)	NS
Brainstem syndrome	28 (14.5%)	14 (15.9%)	NS
Cerebellar syndrome	29 (15.0%)	13 (14.8%)	0.86
Muscle weakness due to injury of the motor tract	25 (13.0%)	6 (6.8%)	0.53
Sensory disturbance	79 (40.9%)	37 (42.0%)	0.74
Other/unknown	18 (9.3%)	11 (12.5%)	
Multifocal symptoms; *n* (%)	24 (12.4%)	12 (13.6%)	0.80

MS: multiple sclerosis; DMT: disease-modifying therapy; ARR: annualized relapse rate; EDSS: Expanded Disability Status Scale; NS: non-significant; NA: not applicable. Percentages are based on non-missing data. Patients could have more than one onset symptom. Asterisks indicate statistical significance (*p < 0.05, **p < 0.01, ***p < 0.001). Missing data proportions are shown in brackets.

### Reasons for delayed DMT initiation and no DMT initiation during follow-up

The most common reason for a delayed or no DMT initiation was patient refusal (100 patients, 35.6% of all) (Table 3). There was no significant difference in refusal rate between the subgroups. Pregnancy-related reasons were significantly more common in the delayed DMT group than in those with no DMT use recorded (19.3% vs 3.1%, respectively; *p* < 0.0001), and the opposite was found for no disease activity, which was more common in those with no DMT initiation during follow-up (35.8% vs 14.8%, respectively; *p* = 0.0003). Among presenting symptoms, optic neuritis was the only symptom with repeated statistically significant differences between treatment groups. It was more common among those with delayed or absent DMT initiation, especially among patients whose treatment was not started due to perceived lack of disease activity.

**Table 3.** Reasons for no DMT initiation or delayed DMT start during follow-up.

	No DMT (*n* = 193)	Delayed DMT (*n* = 88)	*p*-Value
Refusal	68 (35.2%)	32 (36.4%)	1.000
No disease activity	69 (35.8%)	13 (14.8%)	0.0003***
Pregnancy-related	6 (3.1%)	17 (19.3%)	<0.0001***

MS: multiple sclerosis; DMT: disease-modifying treatment. Statistical significance of *p*-values is indicated as follows: ***p < 0.001.

### Evolution of delayed or no DMT use in time

**Figure 2.**
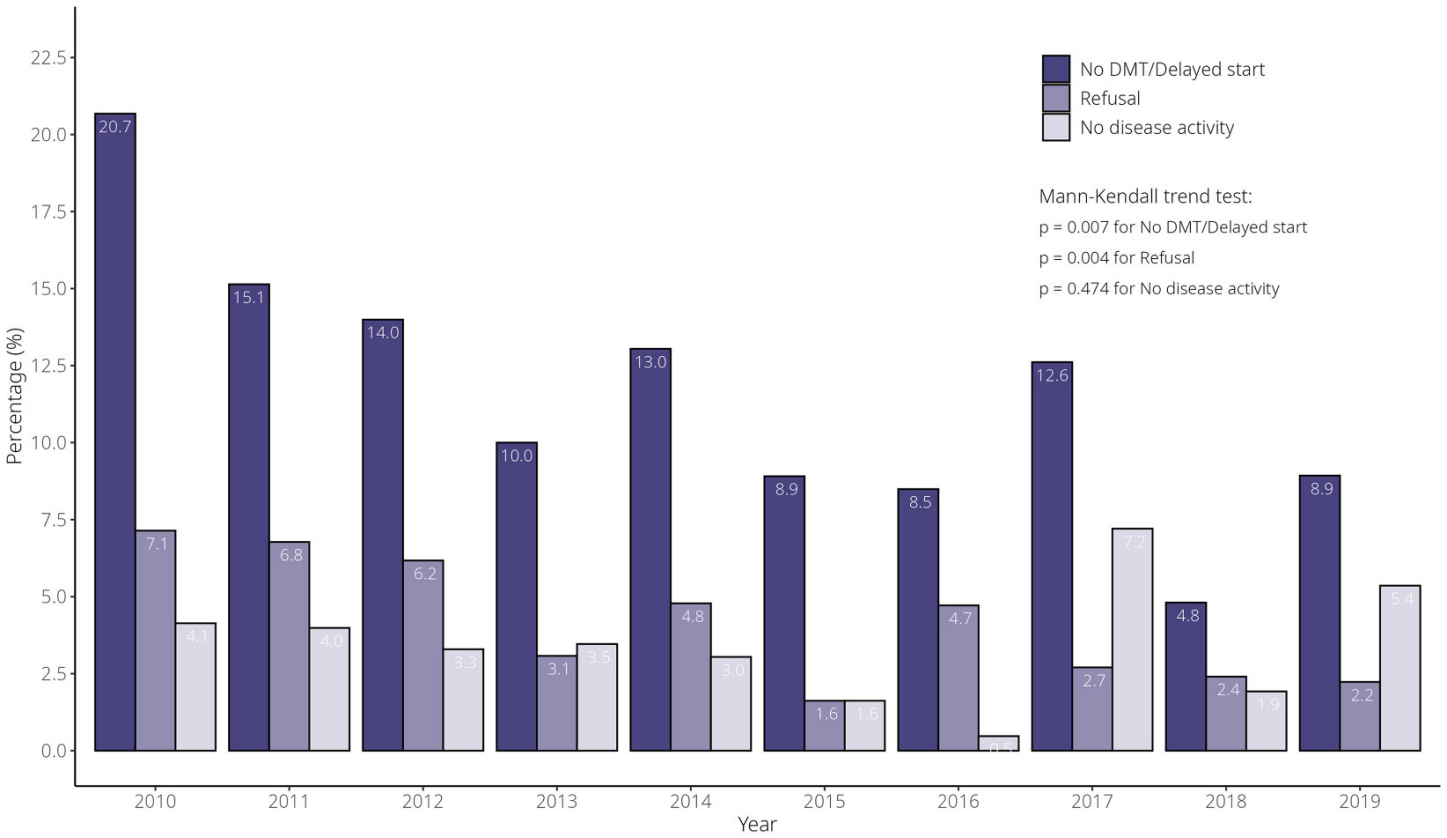
The proportion of patients with no DMT use or over 2-year delayed initiation relative to MS diagnosis annually for 2010–2019. The bar chart illustrates the percentage of patients in three categories over time: (1) no DMT use or delayed DMT initiation (dark purple), (2) refusal of DMT (medium purple), and (3) no documented disease activity (light purple). Statistical analysis using the Mann–Kendall trend test shows a significant downward trend for patients with no DMT use or delayed DMT initiation (*p* = 0.007) and for those refusing DMT (*p* = 0.004). No significant trend was observed for patients with no documented disease activity (*p* = 0.474). The percentages are computed over the whole cohort (MS: multiple sclerosis; DMT: disease-modifying treatment).

We then assessed the evolution of the proportion of pwRRMS with delayed DMT initiation or no use of a DMT during follow-up for the study period 2010–2019 in comparison to the number of new MS diagnoses recorded in the Finnish MS Registry. For the whole study period, a total of 18% of pwRRMS had a delayed initiation or no use of a DMT. We saw a decreasing trend, from 20.7% of patients in 2010 to 8.9% of patients in 2019 (*p* = 0.007, Mann–Kendall trend test; Figure 2). Also, treatment refusal as the underlying cause of delay in DMT initiation/no DMT initiation significantly decreased during this period (7.1% in 2010 to 2.2% in 2019, *p* = 0.004 for the trend).

## Discussion

In this retrospective national cohort study, we identified 281 pwRRMS who had never initiated a DMT or for whom the initiation was delayed by at least 2 years from diagnosis. Patients with delayed treatment initiation or no DMT recorded were older and had longer time from onset symptom to diagnosis compared with early DMT initiators. No DMT initiation was associated with older age but similar disease activity at the time of diagnosis compared to those with delayed DMT initiation. Corticosteroid-treated relapses during the year preceding MS diagnosis were more frequently observed among early DMT initiators (34.2%) compared to those with no DMT (18.7%), delayed DMT initiation (13.6%), or treatment refusal (20.0%). The subgroup with no signs of disease activity had the lowest proportion of corticosteroid-treated relapses (fewer than five individuals), supporting the interpretation that clinical severity influences treatment decisions. Among baseline symptoms, optic neuritis was more frequently observed in patients with delayed or no DMT initiation, particularly among those who did not receive treatment due to perceived lack of disease activity. The most common reason for delayed DMT initiation or not initiating a DMT at all was refusal. Delayed DMT initiation or no DMT during follow-up showed a decreasing trend between 2010 and 2019. There was also a decreasing trend for treatment refusal during 2010–2019 as the underlying reason for the lack of DMT (*p* = 0.004).

Corticosteroid-treated relapses during the year preceding MS diagnosis were significantly more common among early DMT initiators (34.2%) than among patients with no DMT (18.7%), delayed initiation (13.6%), or treatment refusal (20.0%). The group with no signs of disease activity had the lowest rate of corticosteroid-treated relapses (fewer than five individuals). These findings support the interpretation that higher clinical disease activity is a key factor influencing both physician and patient decisions to initiate DMT early.

Patients initiating DMT during the second year after diagnosis were excluded to enable a clear contrast between prompt (within 1 year) and clearly delayed (over 2 years) treatment initiation. The second-year group was considered too heterogeneous for primary analyses.

There is limited knowledge of the magnitude of delayed DMT initiation for pwRRMS. In a Swiss cohort study investigating patients diagnosed between 1996 and 2017, DMT initiation delay over 1 year from diagnosis was seen for 23% of the patients.^11^ In a multicenter study published in 2020, a total of 6.5% of MS patients in the United States, a total of 16.4% in Germany and a total of 36.1% in the United Kingdom had not received a DMT 4.5 years after diagnosis.^12^ It is however important to note also here that not all pwRRMS are recommended a DMT at diagnosis, the proportion of which was reported to be 22.9% of all newly diagnosed patients during 2015 to 2018 in Canada.^13^ In our national registry data, the proportion of newly diagnosed pwRRMS with a delayed DMT initiation or no DMT ever in use was 18% during 2010 to 2019. Our findings align with previous reports.

In a French cohort study, a total of 9.8% of pwMS had not started a DMT within 5 years from diagnosis, and the most common reason for this was lack of disease activity as assessed by the treating neurologist (for 65.8% of patients).^14^ This corresponds well to our findings. Patient preference of no DMTs in Germany and the UK was reported to be only 4%^15^ which is much lower than in our results, possibly owing to differences in patient selection to the studies.

Current McDonald's diagnostic criteria in use since 2017 allow for RRMS diagnosis to be made after a single clinical episode.^16^ Attributable to this, the time from MS diagnosis to the initiation of the first DMT has shortened since 2017.^17,18^ The decreasing trend of delayed DMT initiation or no DMT use can most likely be primarily explained by this change in diagnostic criteria. Also, the number of DMTs available has increased extensively, thus likely also increasing odds of finding a suitable DMT for the patient. For example, availability of oral therapies in addition to injectable or infusion DMTs can have increased compliance, as 42% of patients stated oral DMTs as their preferred medication choice in a study in Germany and the UK.^15^

The gradual decrease in the proportion of patients with delayed or no DMT initiation throughout the study period likely reflects multiple contributing factors, including the broader availability and earlier use of DMTs. Although the 2017 revision of the McDonald diagnostic criteria formally enabled earlier diagnosis of RRMS, we did not observe a sharp inflection in DMT initiation patterns immediately following 2017. A recent nationwide study from Finland^19^ suggested that the implementation of the 2017 criteria was gradual, with noticeable effects emerging only after a delay of a few years, likely due to variation in clinical adoption.

Factors influencing the decision to delay DMT initiation include older age (per additional 5 years OR 1.18, 95% CI 1.09–1.29).^11^ In previous studies common reasons for DMT refusal have included fears related to DMTs, particularly injectables, distrust in pharmaceutical companies and healthcare, pregnancy-related reasons, lack of remaining symptoms, and denial of illness.^13,20–23^ Patients who have never had DMTs can be more concerned about side effects than relapses, and their understanding of the disease's progression may be incomplete in the first year after diagnosis.^24,25^ In some studies, the cost of medication has been a significant reason for the delay in starting therapy.^21–26^ The cost of medication may have influenced the use of MS medications in Finland up until 2012, but since then, MS medications have been fully reimbursed, and costs no longer impact their use. In our retrospective study, the reasons behind patients’ refusal remain unclear, and we do not know to what extent the decisions to refuse are influenced for example by patients’ perceived lack of information, comorbidities, and socioeconomic status.

The included wellbeing service counties were Helsinki and Uusimaa, Southwest Finland, Northern Ostrobothnia, Vaasa, Northern Savonia, and Pirkanmaa. According to national registry data, these six districts represent approximately 67% of all people with MS recorded in the Finnish MS Register.^9^ The included regions span both urban and rural areas and are geographically dispersed across the Southern, Western, and Eastern parts of Finland, covering major population centers such as Helsinki, Tampere, and Turku. Although we did not have access to incidence data or full nationwide comparisons for the earliest stages of RRMS, we have no indication that the clinical phenotype or patient demographics would substantially differ across regions. Thus, we consider that our cohort offers a valid and representative sample of the Finnish MS population.

The strength of our study is that the population-based nationwide data was collected and reviewed in detail by expert neurologists from the participating neurology clinics. This allowed us to focus on pwRRMS and ruling out SPMS or other disease courses likely affecting the DMT initiation decisions. Our study has some limitations. Because we collected real-world observational data, follow-up time and frequency of visits varied between patients. Our study was furthermore not able to address the outcome of delayed DMT initiation on MS prognosis due to lack of reliable follow-up data. If a patient did not initiate DMT, their follow-up was often discontinued at the neurology outpatient clinics in the study, resulting in incomplete follow-up data for patients without medication. One methodological consideration is that, due to the real-world nature of our dataset, in which the majority of patients initiated DMT within one year of diagnosis, we did not employ time-to-event analysis methods such as survival modelling. The number of patients with clearly delayed DMT initiation was limited, and variation in local service structures may have influenced the exact timing of treatment within the early initiation group. Therefore, we chose a stratified, group-based approach to enable a clearer interpretation of clinical and demographic factors associated with early, delayed, or absent treatment initiation.

Future prospective observational follow-up studies are needed to give more information on the clinical effects of delayed DMT initiation. In the future, more in-depth studies into the underlying reasons for DMT refusal are needed. In future studies with larger datasets, it would also be valuable to investigate how temporal changes in patient demographics and clinical characteristics may have contributed to the observed decline in delayed or refused DMT initiation.

In conclusion, delayed DMT initiation in RRMS was influenced by disease activity, patient age, and treatment refusal. The observed decline in delayed or absent DMT initiation from 2010 to 2019 likely reflects advances in diagnostic criteria and treatment options. Further studies are needed to explore the underlying causes of treatment refusal and its impact on MS progression.

## Data Availability

Due to general data protection regulation (GDPR), individual identifiable patient data of the study cannot be shared.
